# Systemic PCSK9 elevation characterises autoimmune liver disease across sexes

**DOI:** 10.1038/s41598-025-28881-y

**Published:** 2025-11-21

**Authors:** Patricia Mester, Vlad Pavel, Petra Stoeckert, Martina Müller, Hauke Christian Tews, Christa Buechler

**Affiliations:** Department of Internal Medicine I, Gastroenterology, Hepatology, Endocrinology, Rheumatology, Immunology, and Infectious Diseases, University Hospital Regensburg, 93053 Regensburg, Germany

**Keywords:** Autoimmune liver disease, Autoimmune hepatitis, Primary biliary cholangitis, Primary sclerosing cholangitis, Sex, Fibrosis score, Biomarkers, Diseases, Gastroenterology, Immunology

## Abstract

Proprotein convertase subtilisin/kexin type 9 (PCSK9) is a key regulator of serum cholesterol. Its expression is particularly abundant in hepatocytes, yet its role in autoimmune liver diseases remains unclear. Here we investigated serum PCSK9 levels in patients with autoimmune liver diseases and compared them to healthy controls, with attention to sex-specific differences. Serum PCSK9 levels were measured in 100 patients with autoimmune liver diseases — 57 with primary sclerosing cholangitis (PSC), 33 with primary biliary cholangitis (PBC), and 10 with autoimmune hepatitis (AIH)—and 88 healthy controls. Subgroup analyses were conducted based on sex and disease type. Diagnostic performance was evaluated using receiver operating characteristic (ROC) curves. PCSK9 levels were significantly elevated in patients with autoimmune liver diseases compared to healthy controls (*p* < 0.001). In male patients, serum PCSK9 levels discriminated between patients with PSC and controls. In female patients, they discriminated between patients with PBC and controls. The area under the ROC curve (AUROC) for distinguishing between these groups was 0.765 ± 0.057 and 0.834 ± 0.047, respectively. Patients with almost normal aminotransferase and cholestasis marker levels (*n* = 47) had significantly higher PCSK9 levels than controls. The AUROC was 0.788 ± 0.039 and a serum PCSK9 level of 224 ng/ml had a sensitivity of 92% and a specificity of 60% for diagnosing autoimmune liver disease. Serum PCSK9 levels did mostly not correlate with serum cholesterol, markers of liver disease severity, the model for end stage liver disease score, or fibrosis stage. Patients who experienced decompensation or required a liver transplant during the course of their disease had PCSK9 levels similar to those who did not experience these adverse events. Serum PCSK9 levels are elevated in both male and female patients with autoimmune liver diseases, independent of cholesterol levels or fibrosis stage. PCSK9 may serve as a biomarker in the diagnosis of autoimmune liver disease, even in patients with almost normal liver function test results.

**Supplementary Information:**

The online version contains supplementary material available at 10.1038/s41598-025-28881-y.

## Introduction

Proprotein convertase subtilisin/kexin type 9 (PCSK9) induces the degradation of the low-density lipoprotein receptor (LDLR), thereby reducing hepatocyte uptake of cholesterol and increasing serum cholesterol levels^1^. Notably, inflammation is associated with increased PCSK9 levels in the serum, whereas cholesterol levels decline^2–5^. Hepatitis C virus infection causes hepatic and systemic inflammation, and effectively eliminating the virus lowers systemic inflammation and PCSK9 levels. In contrast, this is linked with an increase in serum cholesterol levels^6,7^. This illustrates that the association between PCSK9 and cholesterol levels is disturbed in inflammatory diseases^2–4^.

The liver is primarily responsible for synthesising PCSK9 and metabolising lipids, and both processes are affected in chronic liver injury^8–12^. Common causes of chronic liver disease include metabolic dysfunction-associated steatotic liver disease (MASLD), alcohol consumption, and viral infections. All of these liver diseases are associated with increased serum PCSK9 levels^10,13^. While serum cholesterol levels of hepatitis B and C infected patients are reduced, patients with alcoholic liver disease have almost normal cholesterol levels, and patients with MASLD mostly suffer from hypercholesterolemia^14^, showing that higher PCSK9 levels do not necessarily correlate with increased serum cholesterol. In liver cirrhosis, serum cholesterol levels decline^14,15^. It should be noted that serum PCSK9 levels are also low in patients with advanced liver fibrosis, due to impaired PCSK9 release by the injured liver^6,16^.

Experimental evidence supports that PCSK9 plays a role in liver injury. Notably, PCSK9 knockout mice have been shown to be more prone to developing severe hepatic steatosis and fibrosis when fed a high-fat diet^17^. Mijiti et al. demonstrated that blocking hepatic PCSK9 expression protected mice from metabolic steatohepatitis^18^. Humans with genetic inhibition of PCSK9 are not at an increased risk of developing MASLD^19^, and the association of PCSK9 with liver disease in patients with common causes of liver injury remains unclear^1,10,20^.

There is evidence to suggest that patients suffering from autoimmune diseases exhibit changes in their serum PCSK9 levels, which also have a role in disease development. Patients with rheumatoid arthritis have been found to have elevated serum PCSK9 levels, which are associated with disease activity and treatment response^21^. PCSK9 inhibition reduces the risk of systemic lupus erythematosus, yet simultaneously increases the risk of Crohn’s disease^22,23^. A previous study found that patients with active ulcerative colitis had higher serum PCSK9 levels than those with inactive disease^24^. Patients with ulcerative colitis also had higher serum PCSK9 levels than healthy controls^25^. However, our recent study cohort of patients with inflammatory bowel disease (IBD) was found to have PCSK9 levels within the normal range^26^. Consistent with our findings, higher expression of the PCSK9 gene is not associated with IBD^27^.

Primary sclerosing cholangitis (PSC) is a rare, progressive liver disease, and most patients diagnosed with this condition also have underlying IBD^28,29^. Patients with PSC may have elevated cholesterol levels^30^. Those with cholestatic liver diseases are often treated with ursodeoxycholic acid, which lowers serum cholesterol levels^30,31^. Accordingly, similar serum cholesterol levels in patients with IBD, PSC, and healthy controls have been reported^32^. PSC is believed to be an autoimmune disease affecting the biliary tract. It occurs more often in men^33^. Further autoimmune liver diseases are also rare but tend to affect females more commonly^31,34^. Primary biliary cholangitis (PBC) is a progressive, cholestatic, autoimmune liver disease involving the destruction of intrahepatic bile ducts, portal inflammation, and scarring^35^. Unlike PSC, which has a male preponderance, PBC almost exclusively affects women^35,36^. In the context of cholestasis, reduced bile acid synthesis results in impaired intestinal cholesterol uptake and lower biliary cholesterol excretion^37^. Elevated LDL has been reported in both the early and late stages of PBC, and almost all PBC patients present elevated LDL-levels^37,38^. Autoimmune hepatitis (AIH) is a rare liver disease that mostly affects women^39^. AIH patients may also exhibit dyslipidemia, with elevated cholesterol levels documented in almost 50% of cases^14^.

Rare diseases are often inadequately studied, resulting in significant research gaps and limitations that hinder progress in improving outcomes for those affected. These diseases are difficult to diagnose and there are no effective therapies^40^.

PCSK9 is a key regulator of circulating cholesterol and elevated levels have been reported in autoimmune diseases^21,22^. To our knowledge, the levels of PCSK9 in the serum of patients with autoimmune liver diseases have not been studied in detail. This study aimed to determine whether serum PCSK9 levels are increased in patients with the rare autoimmune liver diseases AIH, PBC, and PSC, and to assess whether PCSK9 levels are associated with disease severity, fibrosis stage, or sex. We further explored the potential of PCSK9 as a diagnostic biomarker in these conditions.

## Materials and methods

### Study cohorts

Between 8 December 2021 and 31 January 2025, patients diagnosed with autoimmune liver diseases who were admitted to the University Hospital Regensburg were invited to participate in our study. Those who were pregnant or unable to provide informed consent were excluded from participation. Patients with autoimmune liver disease overlap syndrome were also excluded. Liver disease diagnoses were made following the respective EASL guidelines. Diagnosis of AIH was based on liver parameters, the autoimmune panel, autoantibodies and^41^ a liver biopsy was mostly not required. According to the EASL guidelines^42^ a diagnosis of PBC is confirmed when two out of the following three criteria are met: elevated alkaline phosphatase (AP), either with or without elevated gamma-glutamyl transferase (GGT); the presence of antimitochondrial antibodies, or other specific autoantibodies; and liver biopsy findings of nonsuppurative destructive cholangitis. PSC was diagnosed based on liver parameters (GGT, AP and bilirubin) and magnetic resonance cholangiopancreatography (MRCP) or endoscopic retrograde cholangiopancreatography (ERCP)^43^. During the follow-up period, three patients (two with PSC and one with PBC) required admission to intensive care. One of the patients with PSC did not survive. Blood samples from the patients were collected for analysis of routine laboratory parameters and PCSK9 simultaneously.

The control group comprised students, hospital staff, and spouses of patients. All control subjects were found to be healthy and of normal weight. The study was approved by the Ethics Committee of the University Hospital of Regensburg (Protocol No. 19–1309 − 101, Approval date: 20.02.2019, and Protocol No. 21–2390 − 101, Approval date: 19.05.2021). The study was performed according to the updated guidelines of good clinical practice and the updated Declaration of Helsinki. Written informed consent was obtained from all subjects involved in the study.

### ELISA

The DuoSet ELISA for human PCSK9 (order number DY3888) was acquired from Bio-Techne GmbH (Wiesbaden, Nordenstadt, Germany) and employed in compliance with the company’s protocols. The serum was diluted at a ratio of 1:100. Samples were assessed twice, and the average value was utilized for calculations.

### Liver stiffness measurement

Liver stiffness was assessed in all patients during outpatient visits using acoustic radiation force impulse (ARFI) elastography. Each examination included eight independent measurements taken from different regions of the liver, approximately 5 cm below the liver capsule and 2 cm beneath Glisson’s capsule. The median of these values was used to minimise inter-sample variability. A standardised protocol for patient positioning and breathing, based on established guidelines, was followed throughout the procedure. Imaging and data acquisition were performed using the Logiq™ E10 system (GE HealthCare, Munich, Germany)^44,45^.

### Statistical analysis

The distribution of serum PCSK9 levels was assessed using the Shapiro-Wilk test, which revealed that they were only normal in patients with AIH. warranting the use of non-parametric statistical methods. Group comparisons were performed using the Mann–Whitney U test, Kruskal–Wallis test, and chi-square test as appropriate. For multiple group comparisons, Bonferroni correction was automatically applied within the software environment. Correlations between continuous variables were analysed using Spearman’s rank correlation coefficient. Diagnostic performance was assessed by calculating the Youden index. All analyses were conducted using IBM SPSS Statistics version 26.0 (IBM Corp., Armonk, NY, USA), and a two-tailed p-value < 0.05 was considered statistically significant.

## Results

### Study groups

The distribution of serum PCSK9 levels was assessed using the Shapiro-Wilk test, which revealed normal distribution exclusively in patients with autoimmune hepatitis (AIH) (*p* = 0.731). In contrast, PCSK9 levels deviated from normality in all other groups (controls: *p* < 0.001; PSC: *p* < 0.001; PBC: *p* = 0.013), warranting the use of non-parametric statistical methods.

The study cohort included 88 healthy controls with a median age of 55 (23–86) years, 57 patients with PSC, 33 patients with PBC, and 10 patients with AIH (Table 1). Patients with PSC were the youngest compared to the other patient groups and the controls (*p* < 0.001). The AIH and PBC groups had a higher proportion of females than the PSC group (Table 1). This also differed from the control group (*p* < 0.001 for PBC and *p* = 0.006 for AIH).

The BMI of the control group was not recorded, but none of them were significantly overweight. All of our participants were healthy and reported not suffering from any chronic diseases. Patients with PBC had the highest BMI, which was significantly higher than that of patients with PSC (Table 1). PBC is predominantly diagnosed in females, ^35^ and in the female cohort, patients with PBC also had a higher BMI than patients with PSC (*p* < 0.001).

Patients with PSC exhibited significantly higher levels of alanine aminotransferase (ALT), gamma-glutamyl transferase (GGT) and alkaline phosphatase (AP) compared to those with AIH (Table 1). ALT and GGT levels of patients with PSC were higher than of patients with PBC (Table 1). Total, direct, and indirect bilirubin levels, LDL; cholesterol as well as liver stiffness measurements (FibroScan scores) and the Model of End Stage Liver Disease (MELD) score were comparable across all diagnostic groups.

The prevalence of metabolic comorbidities was inconsistently reported in the medical records and did not differ significantly between cohorts (Table 1).

Most patients with PSC or PBC were prescribed ursodeoxycholic acid, and immunosuppressive medications were mostly given to patients with PSC. The most prevalent adverse outcome during the course of the disease was decompensation, documented in five PSC patients and six PBC patients. Six patients with PSC and three with PBC required a liver transplant (Table 1).

**Table 1:** Details are provided for patients with primary sclerosing cholangitis (PSC), primary biliary cholangitis (PBC), and autoimmune hepatitis (AIH).

Characteristics	PSC	PBC	AIH
Males / Females	31 / 26*** **	2 / 31***	1 / 9**
Age (years)	46 (18–76)**^,^***	61 (31–80)***	62 (24–80)**
BMI (kg/m^2^)	23.0 (16.3–31.8)^40^ **	27.4 (20.2–38.8)^32^ **	23.9 (21.3–35.0)
AST (U/l)	36 (15–177)	27 (20–132)	28 (19–33)
ALT (U/l)	35 (5–205)* *	20 (11–113)*	18 (9–59)*
GGT (U/l)	57 (10–1700)**	42 (11 -1361)*	19 (12–116)**^,^*
AP (U/l)	140 (35–587)**	106 (60–1652)	80 (53–104)**
Bilirubin (mg/dl)	0.7 (0.2–21.3)	0.5 (0.2–9.9)	0.5 (0.2–1.2)
Bilirubin direct (mg/dl)	0.3 (0.1–2.5)^21^	0.2 (0.1–7.3)^30^	0.3 (0.1–0.4)^8^
Bilirubin indirect (mg/dl)	0.5 (0.1–2.8)^21^	0.3 (0.1–2.6)^30^	0.3 (0.1–0.8)^8^
LDL (mg/dl)	121 (50–183)^21^	110 (28–265)^28^	135 (103–163)^8^
Cholesterol (mg/dl)	200 (131–288)^21^	201 (66–359)^28^	207 (170–229)^8^
Fibroscan	1 (0–4)^46^	1 (0–4)	1 (0–4)
MELD score	6 (5–20)^43^	6 (5–28)^30^	7 (6–11)
Diabetes yes/no/n.d	0/5/13	5/23/5	0/8/2
Cardiovascular disease yes/no/n.d.	1/4/13	3/25/5	1/7/2
Hypertension yes/no/n.d	1/4/13	5/25/3	2/6/2
Ursodeoxycholic acid yes/no/n.d.	49/2/6	31/0/2	0/10/0
Immunsuppressive therapy yes/no/n.d	11/40/6	1/32/0	6/4/0
Mayo PSC Risk score high/intermediate/low/n.d.	2/6/19/30	n.d.	n.d.
Decompensation during the disease course yes/no/n.d.	5/23/29	6/27/0	0/10/0
Liver transplantation yes/no/n.d.	6/22/29	3/30/0	0/10/0

Upper-case numbers were used when this measure was unavailable for the entire group.* *p* < 0.05, ** *p* < 0.01, *** *p* < 0.001.

Alanine aminotransferase (ALT), alkaline phosphatase (AP), aspartate aminotransferase (AST), body mass index (BMI), gamma-glutamyl transferase (GGT), low-density lipoprotein (LDL), Model of End Stage Liver Disease (MELD), not defined (n.d.).

### PCSK9 of controls and patients with autoimmune liver diseases

**Fig. 1:**
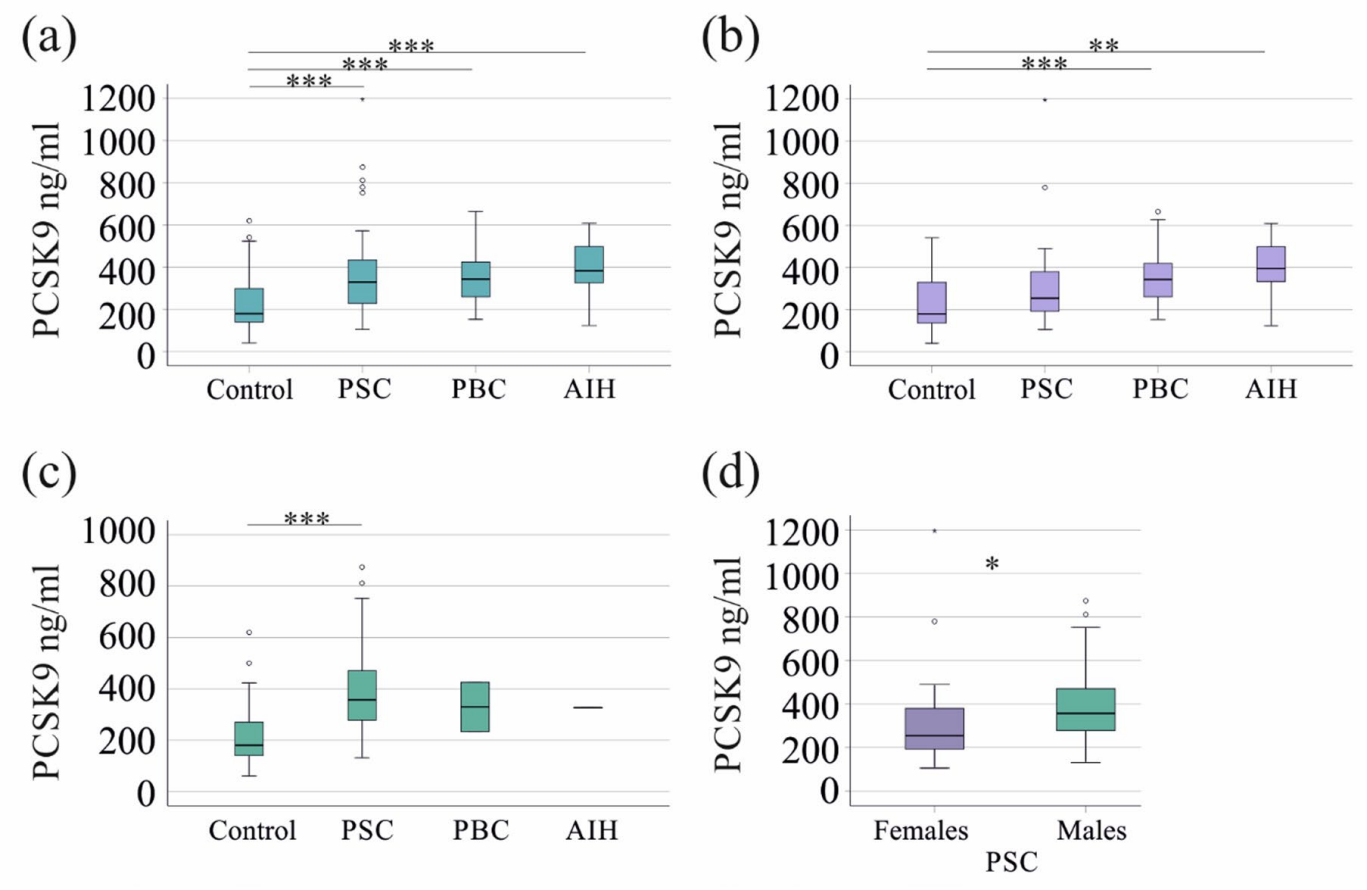
PCSK9 in the serum of patients and controls: (a) Serum PCSK9 levels in controls, patients with primary sclerosing cholangitis (PSC), patients with primary biliary cholangitis (PBC) and patients with autoimmune hepatitis (AIH). (b) Serum PCSK9 levels in female patients and controls. (c) Serum PCSK9 levels in male patients and controls. (d) PCSK9 serum levels in male and female patients with PSC. * *p* < 0.05, ** *p* < 0.01, *** *p* < 0.001.

PCSK9 levels were higher in the serum of all patient cohorts than in the control group (Fig. 1a). The levels were as follows:

- Controls: 180 (40–620) ng/ml.
- PSC: 329 (105–1195) ng/ml.
- PBC: 343 (153–664) ng/ml.
- AIH: 383 (123–609) ng/ml.

In patients with PSC, PCSK9 serum levels were not associated with the Mayo PSC risk score (*p* = 0.665).

A significant difference in serum PCSK9 levels was observed between patients with PSC-IBD and those with isolated PSC, with higher levels detected in the latter group (Figure S1a). PSC patients were older and did not have immunotherapy in comparison to PSC-IBD patients (Table S1). However, serum PCSK9 levels in PSC-IBD patients with and without immunotherapy were similar (*p* = 0.359). PCSK9 positively correlated with direct and indirect bilirubin in PSC-IBD patients. PCSK9 was not associated with cholesterol, LDL, other liver parameters, or the MELD score in both cohorts (Table S2).

### Sex-specific differences in serum PCSK9 levels

Given the strong female predominance in PBC and AIH (up to 90% of patients)^34^, a sex-stratified analysis was performed. In females, serum PCSK9 levels were significantly elevated in patients with PBC and AIH compared to healthy controls. In contrast, PCSK9 levels in healthy females were comparable to those observed in female patients with PSC (Fig. 1b). Among male participants, serum PCSK9 levels were significantly higher in PSC compared to male controls (Fig. 1c). Due to the small number of male patients with PBC (*n* = 2) and AIH (*n* = 1), statistical analysis for these subgroups was not conducted.

Male patients with PSC exhibited significantly higher PCSK9 levels than their female counterparts (Fig. 1d), which was paralleled by a higher body mass index (BMI) (*p* = 0.047) and bilirubin (*p* = 0.038) in males. No significant sex differences were observed in age or other routine laboratory parameters listed in Table 1 (all *p* > 0.05). PCSK9 levels in healthy male and female controls were similar (*p* = 0.718).

### Correlation of PCSK9 with measures of liver health and serum cholesterol

The correlation between serum PCSK9 levels and various factors, including age, BMI, AST, ALT, GGT, AP, total bilirubin, direct bilirubin and indirect bilirubin, as well as the Fibroscan score ranging from 0 (no fibrosis) to 4 (cirrhosis) and the MELD score, was determined in the entire cohort, and because male and female patients with PSC exhibited different serum PCSK9 levels (Fig. 1d), for both sexes. As most PBC and AIH patients were female, the few males were excluded from the analysis.

**Table 2:** Spearman´s correlation coefficients for the correlation of PCSK9 with different parameters.

Characteristics	PSC females	PSC males	PSC males and females	PBC females	AIH females
Age (years)	0.087	0.127	0.076	0.106	0.285
BMI (kg/m^2^)	-0.008	0.207	0.154	-0.120	-0.133
AST (U/L)	-0.391*	-0.011	-0. 090	0.188	-0.218
ALT (U/L)	-0.365	-0.080	-0.98	0.002	-0.345
GGT (U/L)	-0.173	0.021	0.015	0.127	-0.050
AP (U/L)	-0.370	-0.049	-0.116	0.243	-0.159
Bilirubin (mg/dL)	-0.264	0.252	0.123	0.271	-0.037
Bilirubin direct (mg/dL)	-0.096	0.638*	0.493*	0.329	-0.252
Bilirubin indirect (mg/dL)	0.277	0.637*	0.479*	0.210	-0.342
LDL (mg/dL)	-0.286	0.104	-0.039	-0.037	0.216
Cholesterol	-0.333	0.162	-0.019	-0.015	0.393
Fibroscan	-0.011	0.066	0.034	0.158	0.130
MELD Score	-0.063	0.228	0.202	0.372	-0.082

Correlations for PCSK9 in male and female patients as well as the entire primary sclerosing cholangitis (PSC) group, female patients with primary biliary cholangitis (PBC), and female patients with autoimmune hepatitis (AIH). * *p* < 0.05, ** *p* < 0.01.

Alanine aminotransferase (ALT), alkaline phosphatase (AP), aspartate aminotransferase (AST), body mass index (BMI), gamma-glutamyl transferase (GGT), low-density lipoprotein (LDL), Model of End Stage Liver Disease (MELD).

In female patients with PSC, serum PCSK9 was found to be negatively correlated with AST. In male patients with PSC, positive associations with direct and indirect bilirubin were significant (Table 2). No such correlations were detected in female patients with PBC or AIH (Table 2). Correlation analysis showed that PCSK9 did not correlate significantly with laboratory measures of liver health, fibrosis score, the MELD score, serum cholesterol or LDL levels in the different patient cohorts (Table 2).

### Association of PCSK9 and cholesterol with fibrosis

**Fig. 2:**
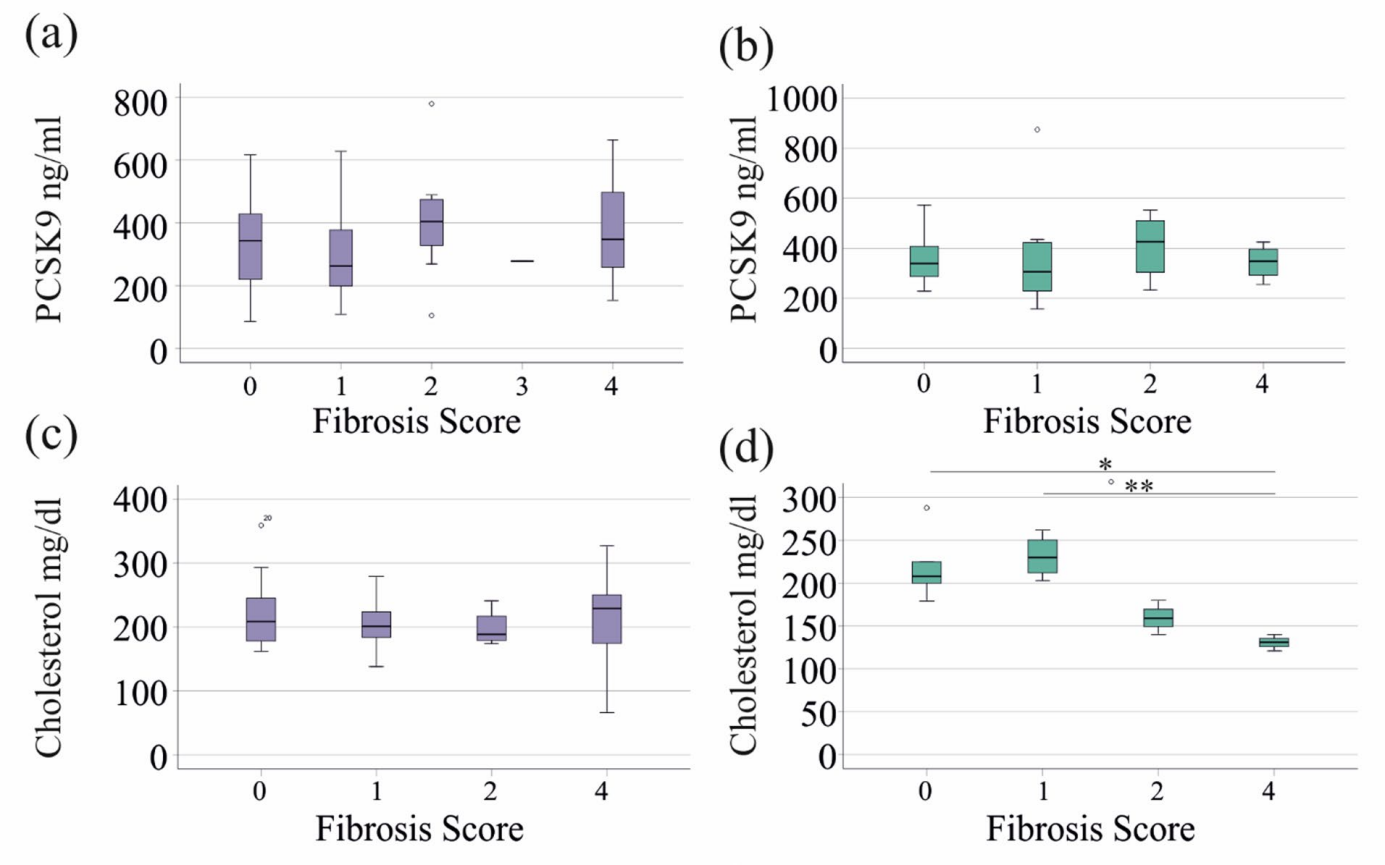
PCSK9 and cholesterol in relation to liver fibrosis: (a) PCSK9 levels in the serum of female patients with primary sclerosing cholangitis (PSC), primary biliary cholangitis (PBC), and autoimmune hepatitis (AIH) in relation to the fibrosis score. (b) PCSK9 in the serum of male patients with PSC, PBC or AIH in relation to the fibrosis score. (c) Cholesterol in the serum of female patients with PSC, PBC or AIH in relation to the fibrosis score. (d) Cholesterol in the serum of male patients with PSC, PBC or AIH in relation to the fibrosis score.

This analysis included female patients with PSC, PBC or AIH. The 20 females without fibrosis, the 27 females with a score of 1, the 7 females with a score of 2, the 1 female with a score of 3, and the 11 females with a score of 4 resembling cirrhosis had similar PCSK9 levels (Fig. 2a).

The association with fibrosis was also analysed in all male patients. The 10 males without fibrosis, the eight males with a score of 1, the five males with a score of 2, and the four males with a score of 4 resembling cirrhosis had similar PCSK9 levels (Fig. 2b).

The serum cholesterol of patients with liver cirrhosis is low^14^, but did not decline in females with higher fibrosis scores (Fig. 2c). However, fibrosis scores 2 and 4 in males were associated with low serum cholesterol levels (Fig. 2d).

### Diagnostic potential of PCSK9 in autoimmune liver diseases

Of clinical relevance, serum PCSK9 levels demonstrated diagnostic utility in distinguishing patients with autoimmune liver diseases from healthy controls. In females with autoimmune liver disease, the area under the receiver operating characteristic curve (AUROC) was 0.618 ± 0.047 (*p* = 0.015). In males, diagnostic performance was higher, with an AUROC of 0.739 ± 0.047 (*p* < 0.001); a cut-off of 215 ng/mL achieved 91% sensitivity and 47% specificity.

**Fig. 3:**
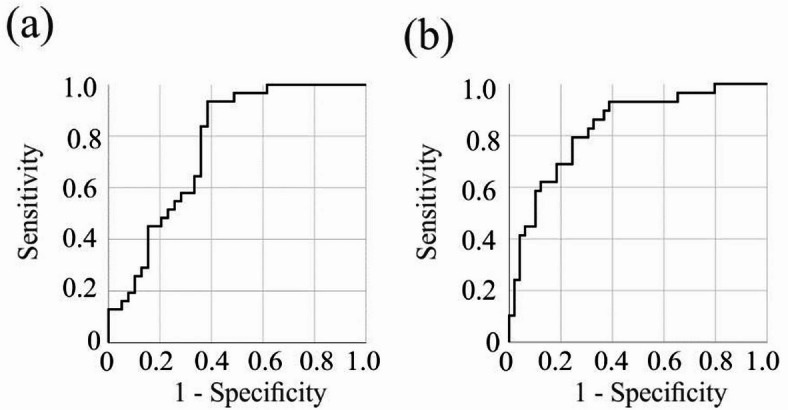
PCSK9 for diagnosing autoimmune liver disease. (a) Receiver operating characteristic (ROC) curve showing the discrimination of female patients with primary biliary cholangitis (PBC) compared to controls. (b) ROC curve showing the discrimination of male patients with primary sclerosing cholangitis (PSC) compared to controls.

In females, the AUROC was 0.765 ± 0.057 (*p* < 0.001) for discriminating between PBC and control groups (Fig. 3a). A cut-off point of 232 ng/mL yielded 94% sensitivity and 62% specificity. However, the AUROC for diagnosing PSC in females was too low at 0.650 ± 0.069 (*p* = 0.044) for an appropriate diagnosis.

In males with PSC the AUROC was 0.834 ± 0.047 (*p* < 0.001), and achieved 93% sensitivity and 61% specificity at a PCSK9 level of 214 ng/mL (Fig. 3b).

Early diagnosis during the course of the disease is warranted. Patients with any autoimmune disease and without fibrosis had higher PCSK9 levels than the control group (*p* < 0.001). The AUROC was 0.755 ± 0.046 (*p* < 0.001) and a PCSK9 serum level of 224 ng/ml had 85% sensitivity and 59% specificity for diagnosing autoimmune liver disease.

**Fig. 4:**
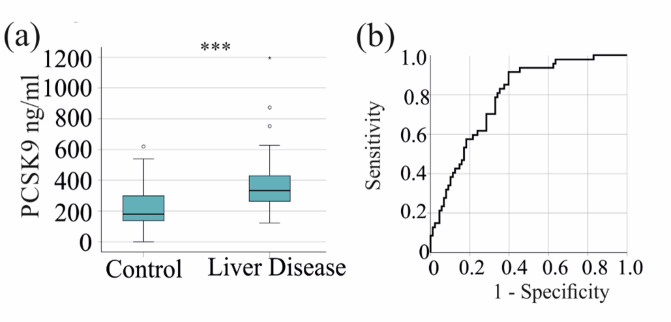
PCSK9 for diagnosis of autoimmune liver disease. (a) Serum PCSK9 levels of controls and patients with AST and ALT < 50 U/L, GGT < 60 U/L and AP < 130 U/L. (b) Receiver operating characteristic (ROC) curve showing the discrimination of patients with AST and ALT < 50 U/L, GGT < 60 U/L and AP < 130 U/L and controls.

Patients with AST and ALT < 50 U/L, GGT < 60 U/L and AP < 130 U/L (the upper limit of normal levels in males) had significantly higher PCSK9 levels than controls (Fig. 4a). The AUROC was 0.788 ± 0.039 (*p* < 0.001) and a PCSK9 serum level of 224 ng/ml had a sensitivity of 92% and a specificity of 60% for diagnosing autoimmune liver disease (Fig. 4b).

### Prognostic potential of PCSK9 in autoimmune liver diseases

**Fig. 5:**
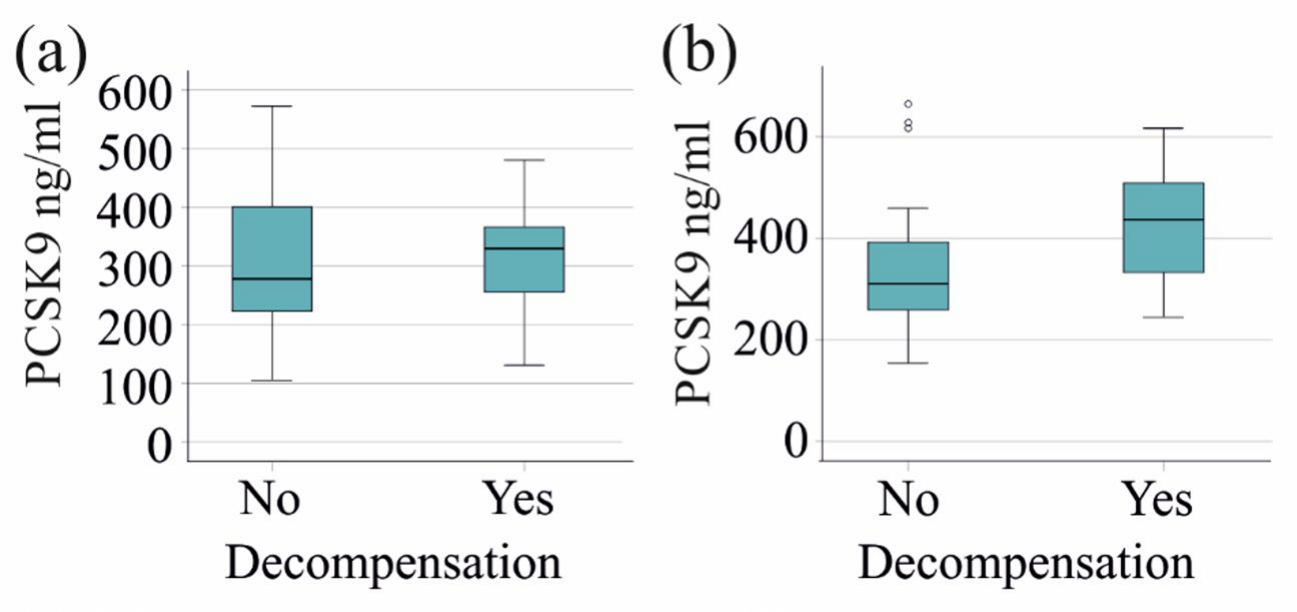
PCSK9 for prognosis of decompensation. (a) PCSK9 levels of patients with primary sclerosing cholangitis without and with decompensation. (b) PCSK9 levels of patients with primary biliary cholangitis without and with decompensation.

PCSK9 levels in the five patients with PSC, as well as in the six patients with PBC who experienced decompensation during the course of the disease, were similar to those in patients who did not experience this adverse event (Fig. 5a, b). The PCSK9 levels of the six patients with PSC requiring liver transplantation did not differ from those of patients without this severe disease progression (*p* = 0.370). The three patients with PBC who underwent transplantation had higher serum PCSK9 levels (*p* = 0.049).

## Discussion

This study demonstrates that serum PCSK9 levels are significantly higher in male patients with PSC and female patients with PBC than in healthy controls, regardless of liver function parameters, cholesterol levels, or fibrosis stage. Notably, PCSK9 levels exhibited strong discriminatory power in identifying patients with autoimmune liver disease despite having almost normal liver parameters. These results imply that PCSK9 could be used as an early diagnostic biomarker for autoimmune liver diseases. Further studies are needed to confirm these results and to investigate the mechanistic role of PCSK9 in hepatic autoimmunity.

PSC is more prevalent in men than women (62%–70% of patients are male) and typically affects individuals in middle age, with a reported median age at diagnosis ranging from 35 to 51 years^36^. The median age at diagnosis was higher in patients with isolated PSC than in patients with PSC-IBD^46^. Indeed, in the current study, PSC-IBD patients were the youngest compared to patients with PSC and all other cohorts, and 60% of these patients were male. PBC typically presents in the mid-to-late 50s, with a female-to-male ratio of 10:1^35^. AIH predominantly affects females, with approximately two-thirds of patients being under 60 years of age and one-third being over 60 years of age^39^. Although we did not collect blood samples at the time of diagnosis, the younger age of patients with PSC-IBD is reasonable. Patients with PBC had the highest BMI, and this was evident across the entire cohort and in females. The cholestasis markers AP and GGT^47^ of our patients with AIH were low compared to the other groups, as expected^48^.

Interestingly, serum PCSK9 levels did not correlate with most of the conventional markers of liver injury, indicating that their elevation in autoimmune liver diseases is unlikely to result from hepatocellular damage alone. A negative correlation of PCSK9 with AST was observed in female PSC patients. In male PSC patients, PCSK9 positively correlated with both direct and indirect bilirubin, but not with total bilirubin, which is the sum of these components. PCSK9 was increased in male but not in female patients with PSC, which is in accordance with positive correlations of PCSK9 and bilirubin in males only. It has been also shown that inhibition of PCSK9 is related with higher direct bilirubin levels in Europeans^49^. Therefore, it is questionable whether the correlations between PCSK9 and AST and bilirubin are of any pathophysiological relevance and will persist in larger cohorts.

Given PCSK9’s central role in cholesterol metabolism, its increase could reflect broader immune or metabolic dysregulation. Although approximately 60% of patients in our cohort exhibited elevated LDL and total cholesterol levels, this is comparable to rates in the general German population of similar age^50^, suggesting that hypercholesterolemia alone does not explain the rise in PCSK9. Moreover, PCSK9 did not correlate with LDL or total cholesterol levels across the patient subgroups. The frequent use of ursodeoxycholic acid in cholestatic conditions, known to lower cholesterol levels^30,31^, also supports a cholesterol-independent mechanism. Further mechanistic studies are warranted to elucidate whether PCSK9 plays a causal role in autoimmune liver pathogenesis or serves as a downstream marker of immune activation.

Hypercholesterolemia is a known cardiovascular risk factor and is frequently observed in patients with autoimmune liver diseases^51,52^. While statins and cholesterol absorption inhibitors are standard lipid-lowering therapies^53^, both have been associated with drug-induced liver injury (DILI) in susceptible individuals^54,55^. Given that PCSK9 inhibitors lower LDL levels without significant hepatotoxicity^56^, they may represent a safer alternative for managing dyslipidaemia in this population. Though higher PCSK9 levels support the rationale for exploring anti-PCSK9 therapy as an option in patients with autoimmune liver diseases at risk for DILI, this warrants further preclinical and clinical investigations.

### PCSK9 and cholesterol levels in the context of liver cirrhosis

Previous studies have shown that circulating PCSK9 and serum cholesterol levels are reduced in patients with liver cirrhosis^6,14–16^. However, this pattern was not observed in female patients with autoimmune liver diseases in our cohort. In male patients with advanced fibrosis (F2 and F4), cholesterol levels were reduced, but PCSK9 concentrations remained elevated, suggesting that PCSK9 regulation is independent of cholesterol metabolism and liver synthetic function.

These findings raise the possibility of sex-specific differences in lipid regulation in the setting of autoimmune liver disease. Serum cholesterol in female patients may be less responsive to hepatic dysfunction than in males. To our knowledge, no prior studies have specifically analysed serum cholesterol dynamics by sex in patients with liver cirrhosis. Our own unpublished data from patients with chronic hepatitis C indicate that both male and female patients with cirrhosis exhibit significantly lower cholesterol levels before and after viral clearance.

Taken together, these observations suggest that in female patients with autoimmune liver diseases, disease-specific mechanisms promoting hypercholesterolemia may override the suppressive effects of cirrhosis on hepatic cholesterol release. Further studies are needed to elucidate the interplay between sex, liver fibrosis, and lipid metabolism in autoimmune liver disorders.

### Diagnostic and prognostic potential of PCSK9

One of the most striking findings of this study is the diagnostic potential of serum PCSK9 levels in distinguishing patients with autoimmune liver diseases from healthy individuals. In female patients with PBC serum PCSK9 levels demonstrated good diagnostic accuracy, with an AUROC of 0.765 ± 0.057. A threshold of 232 ng/mL yielded a high sensitivity of 94%, albeit with moderate specificity (62%), making PCSK9 a potentially useful marker for screening PBC in females. In males with PSC, the AUROC was 0.834 ± 0.047, and achieved 93% sensitivity and 61% specificity at 214 ng/ml PCSK9.

These sex-specific differences in diagnostic performance of PCSK9 in PSC may reflect underlying differences in immunopathology, lipid metabolism, or hepatic PCSK9 regulation. While the clinical utility of PCSK9 as a standalone diagnostic marker remains to be validated in larger, prospective cohorts, our data suggest that it may be a valuable addition to the diagnostic workup.

Early diagnosis during the course of the disease is essential. Patients with any autoimmune disease who do not exhibit fibrosis showed significantly higher levels of PCSK9 compared to controls. The AUROC was 0.755 ± 0.046 (*p* < 0.001), with a serum PCSK9 level of 224 ng/ml demonstrating a sensitivity of 85% and a specificity of 59% for diagnosing autoimmune liver disease. Among the 47 patients with almost normal liver parameters, PCSK9 levels were significantly higher than those of the controls. The AUROC for this group was 0.788 ± 0.039 (*p* < 0.001), and a serum PCSK9 level of 224 ng/ml exhibited a sensitivity of 92% and a specificity of 60% for diagnosing autoimmune liver disease. This shows that serum PCSK9 levels increase even in patients with good liver function, suggesting that increased PCSK9 levels may indicate autoimmune liver disease.

It should be noted that higher serum levels of PCSK9 have also been observed in more prevalent chronic liver diseases, such as hepatitis C virus infection, alcoholic liver disease, and MASLD^1,16^. This suggests that higher serum PCSK9 levels are not specific to rare liver diseases. It is largely unknown whether serum PCSK9 levels in patients with common chronic liver diseases are already increased in patients with almost normal liver parameters.

Moreover, whether serum PCSK9 levels could serve as a biomarker for treatment response remains an open question. As novel immunomodulatory or anti-fibrotic therapies for autoimmune liver diseases are developed^31,57^, monitoring PCSK9 dynamics could offer insight into treatment efficacy and disease activity. However, the current cohort of patients given immunosuppressive therapies had comparable levels of PCSK9 to those not treated with these medications, suggesting that higher PCSK9 levels are not simply a measure of excessive immune response. Future longitudinal studies are needed to explore the predictive and prognostic value of PCSK9 in this context.

The Hepascore, which includes sex, age, bilirubin, GGT, hyaluronic acid and alpha-2-macroglobulin, is highly valuable for estimating overall survival in patients with PSC^58–60^. This score was further shown to be a significant predictor of hepatic decompensation, transplantation, overall mortality, and liver-related mortality in patients with PSC^60^.

The liver outcome score comprises serum prognostic markers such as age, sex, albumin, hyaluronic acid and GGT, and was initially established as a predictor of liver-related death in patients with chronic hepatitis C^61^. This score predicted the need for a transplant or overall death in patients with AIH, PBC, and PSC^60^. In patients with AIH and PBC, this score predicted liver-related mortality and decompensation. In patients with PSC without liver cirrhosis, the Liver Outcome Score was also a predictor of liver-related decompensation^60^. As hyaluronic acid and alpha-2-macroglobulin are not routinely analysed in our patients, these scores were not included. Moreover, almost all of our patients survived.

However, our study found no evidence that serum PCSK9 levels are associated with disease outcome. The Mayo PSC Risk Score estimates transplant-free survival and/or short-term mortality in PSC^62^ and was not related to serum PCSK9 levels. The MELD score can be useful for general prognostic assessment^63^, but it also did not correlate with serum PCSK9 levels. Patients who experienced decompensation or required a liver transplant had similar levels of PCSK9 to patients who did not experience these adverse events. In PBC, these patients had slightly increased levels, but the differences were too small to recommend PCSK9 as a prognostic factor.

One of the most notable characteristics of PSC is its correlation with IBD, as the majority of patients diagnosed with PSC also present with colitis^29^. The serum PCSK9 levels of patients with isolated PSC were higher than those of patients with PSC-IBD. The two cohorts differed in age, with the PSC cohort being older, and in immunosuppressive therapy, with neither being associated with serum PCSK9 levels. A retrospective analysis showed that patients with isolated PSC progress faster to cirrhosis and have a higher Mayo PSC Risk Score^46^. The levels of inflammation markers, such as leukocyte count and C-reactive protein, in both cohorts were similar^46^. PSC is associated with a high prevalence of non-organ-specific autoantibodies, regardless of the presence of IBD^64^. Levels of anti-integrin avb6 autoantibodies in patients with isolated PSC were lower^65^ but the association between autoantibody levels and PCSK9 has not yet been described. Currently, the reason for higher PCSK9 levels in patients with isolated PSC compared to patients with PSC-IBD is unclear.

This observational study was also unable to determine the role of increased serum PCSK9 levels in autoimmune liver disease. Inhibiting PCSK9 has been shown to reduce the differentiation of T helper 17 cells^66^. Various immune cells work together in the development of autoimmune diseases, with T helper 17 cells playing a particularly crucial role in promoting autoimmune inflammation. The number of T helper 17 cells in the peripheral blood is higher in patients with PSC, PBC and AIH than in healthy individuals^67^. Further functional studies are needed to clarify the role of PCSK9 in immune cell responses in autoimmune liver diseases.

The study’s limitations can be attributed to four main factors. Firstly, the number of patients with rare liver disease included in the study was small. Secondly, metabolic comorbidities were not consistently documented in the medical records. Thirdly, PCSK9 was only measured once during the disease course, from serum obtained during an outpatient visit. Fourthly, the laboratory parameters and BMI of the control group were not recorded.

## Conclusions

This study demonstrates that serum PCSK9 levels are significantly elevated in patients with autoimmune liver diseases—PSC, PBC, and AIH—regardless of disease severity, liver function parameters, or fibrosis stage. Importantly, PCSK9 showed good diagnostic performance, particularly in male patients with PSC and female patients with PBC, and may serve as a complementary biomarker in the diagnostic evaluation of autoimmune liver diseases. These findings suggest that PCSK9 is regulated independently of classical liver injury markers and cholesterol metabolism, underscoring its potential role as both a diagnostic and therapeutic target. Future studies should assess the value of PCSK9 as an early detection marker in broader population-based cohorts.

## Supplementary Information

Below is the link to the electronic supplementary material.

Supplementary Material 1

Supplementary Material 2

## Data Availability

The original contributions presented in this study are included in the article/supplementary material. Further inquiries can be directed to the corresponding author.
