# Supplementary material for: Systemic PCSK9 elevation characterises autoimmune liver disease across sexes
Source: Sci Rep. 2025 Nov 21;15:41185. doi: 10.1038/s41598-025-28881-y (PMC12638933; doi:10.1038/s41598-025-28881-y)
Supplement: Supplementary file 1 — Supplementary Material 1 [file 41598_2025_28881_MOESM1_ESM.pdf]

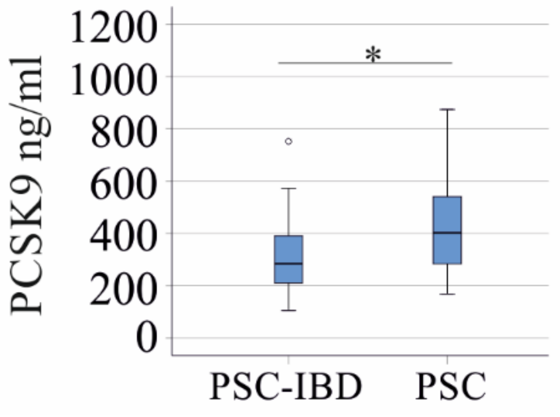

Figure S1. PCSK9 in the serum of patients with primary sclerosing cholangitis (PSC) and inflammatory bowel disease (PSC-IBD) and patients with isolated PSC.

Table S1. Details are provided for patients with primary sclerosing cholangitis (PSC) and inflammatory bowel disease (PSC-IBD), as well as for patients with isolated PSC * $p < 0.05$, ** $p < 0.01$.

Characteristics	PSC-IBD	PSC
Males / Females	24 / 15	7 / 11
Age (years)	43 (18 – 67)	55 (26 – 76)*
BMI (kg/m^2)	23.3 (16.3 - 31.8)	24.0 (18.0 - 29.8)
AST (U/l)	44 (16 -161)	29 (15 - 177)
ALT (U/l)	43 (7 - 205)	31 (5 - 89)
GGT (U/l)	44 (10 - 1700)	73 (11 - 345)
AP (U/l)	140 (35 - 587)	139 (54 - 537)
Bilirubin (mg/dl)	0.7 (0.2 – 21.3)	0.9 (0.3 – 14.0)
Bilirubin direct (mg/dl)	0.4 (0.1 – 2.5)	0.3 (0.1 – 2.2)
Bilirubin indirect (mg/dl)	0.4 (0.1 – 1.2)	0.6 (0.2 – 2.8)
LDL (mg/dl)	117 (50 - 138)	135 (67 - 152)
Cholesterol (mg/dl)	197 (140 - 288)	214 (131 - 279)
Fibroscan	1 (0 - 4)[34]	1 (0 - 4)[12]
MELD score	6 (6 – 20)[29]	7 (5 – 18)[14]
Diabetes yes/no/n.d	2/17/20	0/5/13
Cardiovascular Disease yes/no/n.d.	2/17/20	17/0/1
Hypertension yes/no/n.d	1/18/20	1/4/13
Ursodeoxycholic acid yes/no/n.d.	32/1/6	17/1/0
Immunsuppressive therapy yes/no/n.d	11/22/6	0/18/0**
Mayo PSC Risk score high/intermediate/low/n.d.	1/5/15/18	1/1/4/12
Decompensation during the disease course yes/no/n.d.	4/18/17	1/5/12
Liver transplantation yes/no/n.d.	5/17/17	1/5/12

Alanine aminotransferase (ALT), alkaline phosphatase (AP), aspartate aminotransferase (AST), body mass index (BMI), gamma-glutamyl transferase (GGT), low-density lipoprotein (LDL), Model of End Stage Liver Disease (MELD), not defined (n.d.)

Table S2. Spearman´s correlation coefficients for the correlation of PCSK9 with different parameters in patients with primary sclerosing cholangitis (PSC) and inflammatory bowel disease (PSC-IBD) and patients with isolated PSC. * p < 0.05, ** p < 0.01.

Characteristics	PSC-IBD	PSC
Age (years)	0.127	-0.338
BMI (kg/m^2)	0.324	-0.599
AST (U/L)	-0.212	0.209
ALT (U/L)	-0.127	0.166
GGT (U/L)	-0.117	0.077
AP (U/L)	-0.349*	0.236
Bilirubin (mg/dL)	-0.017	0.268
Bilirubin direct (mg/dL)	0.669**	-0.154
Bilirubin indirect (mg/dL)	0.665**	-0.154
LDL (mg/dL)	-0.009	0.200
Cholesterol	0.096	-0.100
Fibroscan	-0.086	0.370
MELD	0.116	0.433

Alanine aminotransferase (ALT), alkaline phosphatase (AP), aspartate aminotransferase (AST), body mass index (BMI), gamma-glutamyl transferase (GGT), low-density lipoprotein (LDL), Model of End Stage Liver Disease (MELD), not defined (n.d.)
